# High Q Dielectric Titanium Tellurite Thick Films on Alumina Substrates for High Frequency Telecommunications

**DOI:** 10.3390/ma15020467

**Published:** 2022-01-08

**Authors:** Xinming Su, Alexander Tkach, Jerzy Krupka, Paula M. Vilarinho

**Affiliations:** 1 Department of Materials and Ceramic Engineering, CICECO—Aveiro Institute of Materials, University of Aveiro, Campus de Santiago, 3810-193 Aveiro, Portugal; xinming@ua.pt (X.S.); atkach@ua.pt (A.T.); 2 Institute of Microelectronics and Optoelectronics, Warsaw University of Technology, 00662 Warsaw, Poland; j.krupka@imio.pw.edu.pl

**Keywords:** TiTe_3_O_8_ thick films, electrophoretic deposition, alumina substrates, high-Q dielectrics, microwave properties

## Abstract

The vital role of high-quality-factor (Q) high-frequency (f) dielectric resonators in the growing microwave telecommunication, satellite broadcasting and intelligent transport systems has long motivated the search for new, small size, and lightweight integrated components and packages, prepared by low cost and sustainable processes. One approach is replacing the currently used bulk ceramic dielectrics by thick films of low-sintering-temperature dielectrics fabricated by affordable processes. Here we demonstrate the fabrication of high-Q TiTe_3_O_8_ thick films directly on low loss Al_2_O_3_ substrates by electrophoretic deposition using sacrificial carbon layer. Nineteen-micrometre-thick TiTe_3_O_8_ films on Al_2_O_3_ sintered at 700 °C are found to have a relative permittivity ε_r_ of 32 and Q × f > 21,000 GHz. Being thus able to measure and provide for the first time the microwave dielectric properties of these films, our results suggest that TiTe_3_O_8_ films on Al_2_O_3_ substrates are suitable for microlayer microstrip array applications.

## 1. Introduction

Microwave dielectric materials play a vital role within a wide range of applications from terrestrial and satellite communication including software radio, Global Positioning Systems (GPS), and Direct Broadcast Satellite (DBS) television and environmental monitoring via satellites. The recent progress in microwave telecommunication, satellite broadcasting and intelligent transport systems (ITS) has resulted in an increasing demand for dielectric resonators (DRs), which are low loss ceramic pucks used mainly in wireless communication devices [1]. The key properties required for DR materials are high quality factor-frequency product (Q × f), high relative permittivity (ε_r_) and near zero temperature coefficient of resonant frequency (τ_f_) or temperature coefficient of the relative permittivity (TCε_r_). The relative permittivity determines the size of the electronic component, the temperature coefficient of resonant frequency establishes the frequency stability, and the loss tangent (tanδ) or quality factor (Q = 1/tanδ) controls the selectivity and performance of the device [2]. An optimal dielectric resonator that satisfies these three properties simultaneously is difficult to achieve solely in a particular material.

In order to meet the specifications of the current and future systems, improved or novel microwave components based on dedicated dielectric materials and new designs are required [3]. In addition, with the recent evolution in mobile phone and satellite communication systems, using microwaves as the carrier, the research and development in the field of device miniaturization is needed [4]. This fast-growing mobile/wireless communication industry is demanding small size and lightweight integrated components and packages at low cost. A method to meet these requirements, which are now under consideration by the community, is the replacement of the currently used bulk ceramic dielectrics by dielectric thick films [4,5].

Electrophoretic deposition (EPD) is one of the processing techniques of thick films. EPD is a colloidal process, in which charged particles dispersed in a stable suspension are driven by electric field to move towards oppositely charged electrodes to build up a particulate coating by deposition on one of the electrodes [3,6,7,8,9,10]. The main advantages of EPD are related to low cost, versatility and ability to coat conformal substrates. However, the utilization of insulating substrates required for low loss microwave applications raises difficulties for the preparation of continuous thick films by EPD, since to apply the electric field during the EPD process the substrate should be a conductor. Moreover, a high corrosion resistance in the EPD suspension and thermal stability are two major criteria that also limit drastically the use of conductive substrates in EPD [7]. This last aspect is of particular relevance when the thick film sintering temperature is high (>1000 °C), thus, restricting considerably the choice of electrodes to high temperature conductive oxides or noble metals, such as gold, platinum and palladium; both expensive alternatives.

There is, however, an approach developed for preparation of thick films by EPD on poorly conducting substrates, using a fugitive layer of graphite [11,12]. Moreover, Y-stabilized zirconia (YSZ) films deposited this way on NiO-YSZ for fuel cell application were reported to be of improved quality. In our previous work, we adapted this approach to the more critical case of EPD films on insulating dielectric substrates (as alumina and glass) for electronic applications [13]. Thin sacrificial layers of conducting carbon were coated onto non-conducting alumina substrates to facilitate the conduction on the substrate surface. The carbon coating burns out during the sintering step, not interfering with the final product. To prove the concept, a layer of high Q ceramic powders of BaNd_2_Ti_5_O_14_ (BNT) was deposited by EPD on the top of the carbon coated alumina. One-hundred-micrometre-thick BNT films on Al_2_O_3_ substrates exhibited ε_r_ and Q of 146 and 1161 at about 10 GHz when sintered at 1250 °C/1 h. Besides high Q values BNT films on alumina possess high thermal stability and, therefore, are potentially useful for high frequency applications [13]. To date this is the only report on EPD to produce high-Q dielectric thick films on alumina substrates. A limitation of the BaO–Nd_2_O_3_–TiO_2_ system is the high sintering temperature (>1300 °C) required to densify the ceramic bulk or thick layers.

Looking for low sintering temperature dielectrics with low dielectric losses and high capacitance stability tellurium-based systems stand out [14,15,16,17,18,19,20,21,22,23,24,25,26,27,28,29]. Ceramics of Te-based compounds sinter at temperatures under 900 °C [28,29] and exhibit low dielectric losses (Q × f up to 66,000 GHz for Zn_2_Te_3_O_8_ ceramics [23]) and dielectric permittivities ranging from 10.5 for MgTe_2_O_5_ [25] to 56 for Bi_2_TeO_6_ [16]. The temperature coefficient of resonant frequency varies from −119 ppm/°C for TeO_2_ [16] to +372 ppm/ °C for BaTiTe_3_O_9_ [18]. For well sintered TiTe_3_O_8_ ceramics, ε_r_ is of 50 and Q × f is of 30,600 GHz at a measurement frequency of 5 GHz [15]. However, there is only one report on the preparation of tellurium-based thick films by EPD [30]. In this paper we reported the low-frequency dielectric properties of the TiTe_3_O_8_ films on platinized silicon [30]. Moreover, so far there is no report on the microwave characterization of Te-based thick films neither on their deposition on alumina.

In the present work, we have extended our previous approach of using sacrificial carbon layers for EPD on fully non-conducting substrates, to fabricate for the first time low sintering temperature high Q dielectric Te based thick films on insulator Al_2_O_3_ substrates. The dielectric characterization at high frequency suggests that these films are suitable for microlayer microstrip arrays.

## 2. Materials and Methods

The work here described comprises the following experimental steps: preparation of a stable suspension of TiTe_3_O_8_ particles synthesised by solid state reaction, carbon coating of the nonconducting Al_2_O_3_ substrates, EPD of green TiTe_3_O_8_ thick films on carbon coated Al_2_O_3_ substrates and their sintering, as well as electrical characterization of TiTe_3_O_8_ thick films on Al_2_O_3_ at microwave frequencies.

TiTe_3_O_8_ powders were synthesized from reagent-grade TeO_2_ (>99%, Sigma-Aldrich, Saint Louis, MO, USA) and TiO_2_ (>99%, Merck KGaA, Darmstadt, Germany) via a conventional solid-state-reaction method, as per [30]. The starting precursors were weighed according to the molar ratio of 3:1, ball mixed with ethanol and dried at 70 °C for 5 h. The mixed powders were calcined at 620 ºC for 50 h and milled for 5 h in a planetary ball mill at 200 rpm using Teflon pots and zirconia balls. Milled TiTe_3_O_8_ powders with particle size below 10 μm were ultrasonically dispersed in acetone (>99.5%, Panreac Química SLU, Castellar del Vallès, Spain) with a concentration of 10 g/l. Triethanolamine (TEA) (>99%, Merck KGaA, Darmstadt, Germany) was added as a suspension stabilizer to favour the EPD process [31]. The addition of TEA increased considerably the zeta potential of the suspension to 46.5 mV at pH of 8.1 that guaranteed the fabrication of crack free and homogeneous films. The suspensions were magnetically stirred for 5 h at room temperature. The stability of the suspensions was analysed by transmittance of the UV light (UV-2101/3101PC, Shimadzu Corporation, Kyoto, Japan), particle size distribution and zeta potential techniques (Malvern Zeta sizer Nano ZS, Malvern, Worcestershire, UK).

Non-conducting alumina substrates were coated with a sacrificial conducting carbon film that acts as a temporary electrode being burned at high sintering temperatures without leaving any residual contaminations, as mentioned before. Following our previous work [13], a radio frequency (RF) magnetron sputtering (CRIOLAB, Porto, Portugal) was used to perform the deposition of the carbon layer from the corresponding target (purity > 99.9%, Sofacel Inc., Madrid, Spain) of 55 mm in diameter and 3 mm in thickness on 25.4 × 25.4 × 0.0254 mm^3^ polycrystalline Al_2_O_3_ substrates (99.6%, Coors Ceramics U.K., Glenrothes, Fife, Scotland, UK, with the remaining 0.4 wt. % assumed to be a silicate based sintering aid). The carbon coatings were prepared at a substrate–magnetron distance of 120 mm under Ar pressure of 5.8 × 10^–3^ mbar, with a magnetron current of 0.3 A and a substrate bias of 470 V. The thickness of the carbon layer was controlled by the deposition time.

The carbon coated Al_2_O_3_ was employed as a substrate for the deposition of TiTe_3_O_8_ films and stainless steel was chosen as the counterpart electrode. EPD was carried out for 3 min under a dc voltage of 100 V from the voltage supply (Glassman High Voltage Inc., High Bridge, NJ, USA). The obtained films were dried for more than 24 h at room temperature and then sintered from 680 °C to 720 °C for 5 h in a closed alumina crucible together with TeO_2_ powders, to avoid Te volatilization. The use of TeO_2_ powder was reported to increase the partial pressure of TeO_2_ and to suppress the evaporation from TiTe_3_O_8_ [30].

The crystallographic structure and phase content of TiTe_3_O_8_ films were analysed by X-ray diffraction (XRD, PANalytical X’Pert Pro diffractometer, Philips, Amsterdam, The Netherlands, Cu-Kα radiation, 45 kV and 40 mA), used from 10° to 80° 2θ with a step size of 0.025°. The microstructure of the thick films was observed using scanning electron microscopy (SEM, SU-70, Hitachi, Tokyo, Japan) coupled with energy-dispersive X-ray spectroscopy (EDS, QUANTAX 400, Bruker, Billerica, MA, USA). The thickness of green and sintered films was evaluated by a micrometre and SEM.

For microwave frequency characterization the split post dielectric resonator (SPDR) method was used [32]. Thus, ε_r_ and quality factor (Q) were determined at ~20 GHz. The method is based on measurements of resonant frequency and quality factor of the empty resonator, the resonator with the substrate (Al_2_O_3_), and finally the resonator with dielectric deposited on top of the substrate (in this case TiTe_3_O_8_ on Al_2_O_3_) with the same exact dimensions and shape. The dielectric parameters of the measured thick dielectric films are calculated based on full-wave electromagnetic analysis.

## 3. Results

Before alumina substrates are used for EPD, the essential step is to sputter a carbon layer upon it to make them conductive, as explained before. The carbon coatings certainly affect the EPD process and thereby the quality of TiTe_3_O_8_ films and their final dielectric performance. According to our previous results, there is an optimum carbon layer thickness in the range between 200 and 400 nm that guarantees a continuous uniform high quality film deposition [13]. Carbon layers with thickness below 100 nm cannot be used as temporary electrodes for EPD since the formed layer is not sufficiently conductive to permit the deposition of a continuous film. On the other hand, with too thick (>500 nm) carbon layers, non-conformal and non-uniform films are obtained after sintering, because thick carbon layers restrict the final film adhesion to the substrate. Therefore, ~300 nm thick carbon layer was selected to be used in the present work.

**Figure 1:**
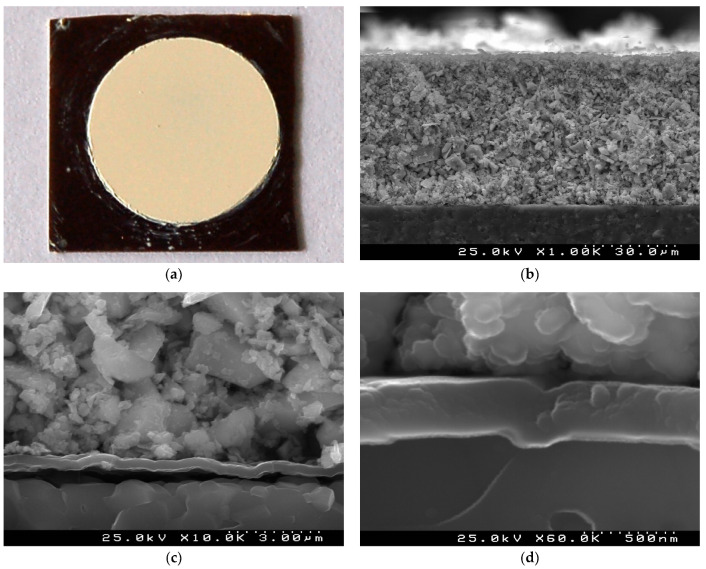
A surface optical image (**a**) and cross section SEM micrographs (**b**–**d**) of a green TiTe_3_O_8_ thick film deposited by EPD under 100 V for 3 min on carbon coated 25.4 × 25.4 × 0.0254 mm^3^ alumina substrate: very uniform and crack free TiTe_3_O_8_ film with a circular shape on a background of a black carbon layer (**a**), average film thickness of ~50 µm (**b**) and compact microstructure of the film (**c**) on a continuous ~300 nm carbon layer (**d**) are visible.

The surface of the green (non-sintered) TiTe_3_O_8_ thick films deposited by EPD under 100 V for 3 min on carbon coated alumina substrate is presented in Figure 1a. Very uniform and crack free TiTe_3_O_8_ film with circular shape is seen on the background of the black carbon layer with square shape. The cross-section microstructure of these green films is illustrated in Figure 1 b,d. The low magnification SEM micrograph depicts a very uniform, compact and conformal ~50 µm thick TiTe_3_O_8_ film on the carbon coated alumina substrate (Figure 1b,c). TiTe_3_O_8_ particles are densely packed and deposited on the substrate thanks to the carbon coating conductive surface. The uniform continuous conformal carbon layer with thickness of ~300 nm can be easily observed from the high magnification micrographs (Figure 1c,d).

**Figure 2:**
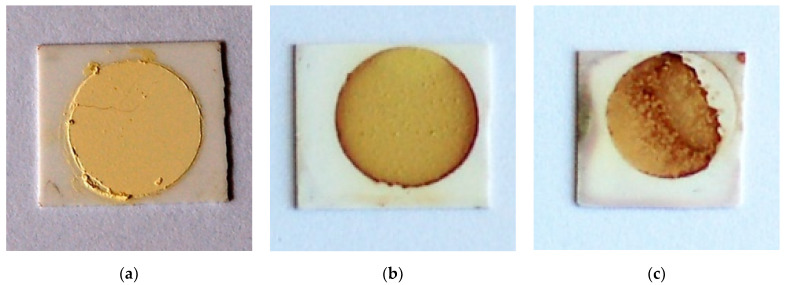
Optical images of TiTe_3_O_8_ films deposited on carbon coated 25.4 × 25.4 × 0.0254 mm^3^ alumina substrates and sintered at 680 (**a**), 700 (**b**) and 720 °C (**c**) for 5 h, respectively. Films sintered at 680 °C are not fully dense and some films sintered at 720 °C exhibit an irregular surface with a reddish colour resulting from a reaction with the alumina substrate.

**Figure A1:**
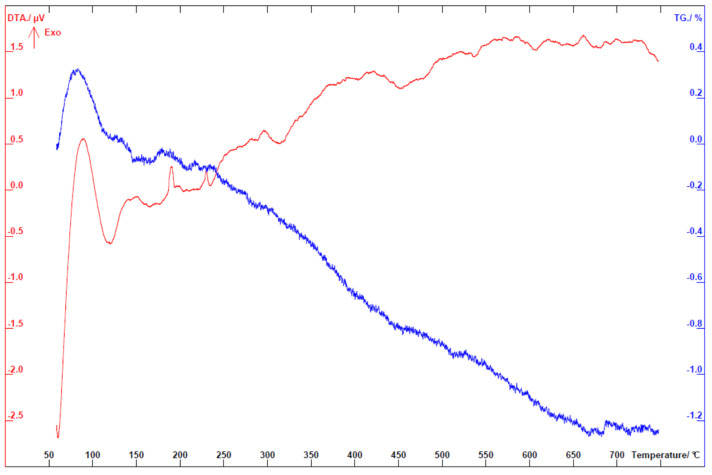
Differential thermal analysis (DTA) and thermogravimetric (TG) analysis curves recorded in air on graphite coated Al_2_O_3_ substrate.

The optical images of TiTe_3_O_8_ films deposited on carbon coated alumina substrates sintered for 5 h in air at 680 (a), 700 (b) and 720 °C (c), respectively, are presented in Figure 2. Sintered TiTe_3_O_8_ films exhibit the yellow colour characteristic of polycrystalline TiTe_3_O_8_ and the alumina substrates present the original white colour. For films sintered at 680 °C (Figure 2a), the substrate is slightly darker than for the other sintered films. This is possibly due to an incomplete burn out of the sacrificial carbon layer, although thermal analysis (see Appendix A Figure A1) clearly indicates that the carbon layer oxidizes in air below 660 °C and thus below the sintering temperature of TiTe_3_O_8_ films. After sintering at 680 °C, films still present a powder-like aspect, similar to the green ones, and do not have good adhesion to the alumina substrates, being easily detached. Therefore, under the present conditions, the sintering temperature of 680 °C is not enough to fully densify TiTe_3_O_8_ thick films on Al_2_O_3_.

Films sintered at higher temperatures, 700 °C and 720 °C, are denser and do not detach from the substrate. However, films sintered at 720 °C exhibit areas with an irregular surface and a reddish colour, as seen from Figure 2c. For films sintered at 700 °C, (Figure 2b) only some reddish vestiges at the edges may be seen. This reddish colour, not observed for the films sintered at 680 °C, may be indicative of some reaction with the alumina substrate. Indeed, in our previous work [33] we proposed that the formation of 1:1 stoichiometry binary Al_2_O_3_-TeO_2_ compound results from the oxidation of TeO_2_, which occurs at temperatures > 600 °C to form Te_4_O_9_ and TeO_3_, that triggers the formation of Al_2_TeO_6_. Under vacuum the oxidation of TeO_2_ does not take place and there is no reaction between Al_2_O_3_ and TeO_2_.

**Figure 3:**
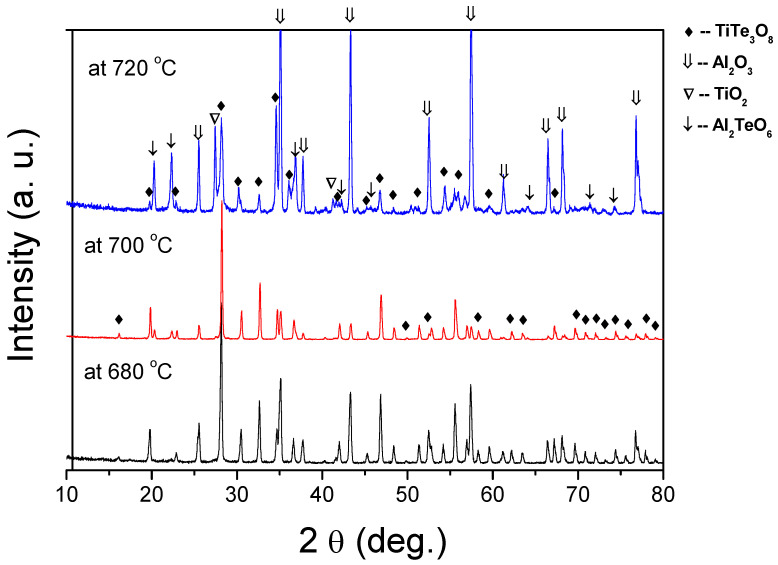
XRD patterns of TiTe_3_O_8_ films deposited on carbon coated alumina substrates and sintered at 680, 700 and 720 °C for 5 h. The main film’s phase is TiTe_3_O_8_. For films sintered above 700 °C Al_2_TeO_6_ is formed due to a reaction between TeO_2_ and Al_2_O_3_ substrates.

To understand what gives rise to these observations and to support our predictions we conducted XRD analysis to examine the evolution of the formed phases under different sintering conditions (Figure 3). For the sintering temperature of 680 °C, the XRD pattern reveals only peaks of TiTe_3_O_8_ phase (JCPDS 70-2439) identical to those of calcined powders [30]. However, as hypothesised, for films sintered at 700 °C and 720 °C, a new phase of Al_2_TeO_6_ (JCPDS 15-0689) can be identified. For films sintered at 700 °C, Al_2_TeO_6_ is residual, but for films sintered at 720 °C, Al_2_TeO_6_ is clearly visible and even peaks of TiO_2_ can be detected, indicative of TiTe_3_O_8_ decomposition. Thus, the reddish colour observed in TiTe_3_O_8_ thick films sintered at temperatures higher than 700 °C is related to the formation Al_2_TeO_6_ phase, which results from the reaction between TeO_2_ from TiTe_3_O_8_ and alumina substrates.

**Figure 4:**
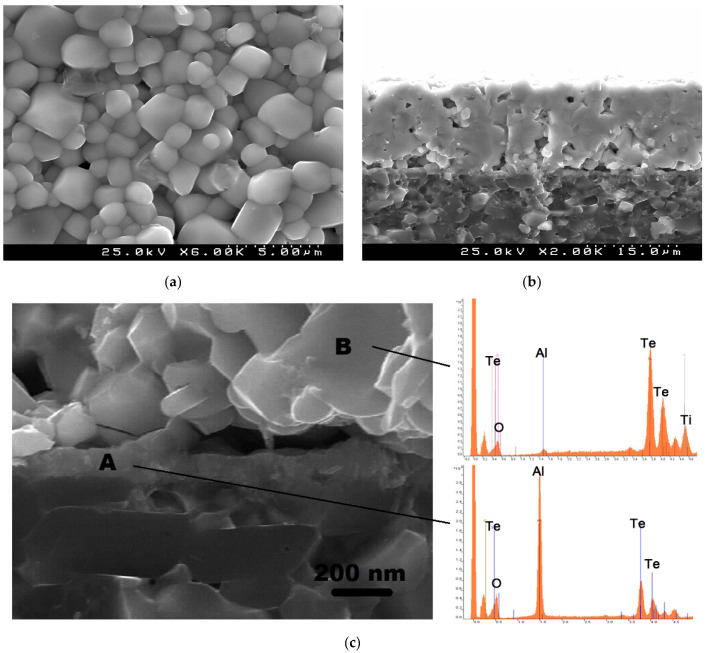
SEM micrographs of TiTe_3_O_8_ films deposited on carbon coated alumina substrates sintered at 700 °C for 5 h, surface (**a**), cross section (**b**) and high magnification cross section and energy dispersive spectra for indicated locations (**c**). Under these sintering conditions (700 °C for 5 h in air) TiTe_3_O_8_ films are dense and uniform with the presence of a thin (<200 nm) reaction interlayer of Al_2_TeO_6_ confined to the interface between the film and the substrate.

We have also inspected the microstructure of the sintered TiTe_3_O_8_ films deposited on carbon coated alumina substrates, for the films sintered at 700 °C for 5 h (Figure 4). Films present a uniform and dense surface microstructure with the typical cubic shaped grains of TiTe_3_O_8_ and well visible grain boundaries (Figure 4a). In agreement, a dense microstructure (with only some residual porosity), a thickness of ~19 μm as well as a good adhesion to the substrate can be observed from the cross-section SEM micrographs (Figure 4b). There is also a thin reaction layer between the film and the substrate interface with a thickness of <200 nm (Figure 4c).

EDS was used for the analysis of the interface between TiTe_3_O_8_ films and Al_2_O_3_ substrates (Figure 4c). The interlayer at the interface between the film and the substrate (region A) is Al and Te rich, indicating the presence of Al_2_TeO_6_ and confirming the previous XRD data. On the other hand, for region B, which corresponds to the main bulk of the thick film, the detected elements are mainly Te and Ti, from TiTe_3_O_8_, being the peak for Al considerably low. Thus, under the sintering conditions of 700 °C for 5 h in air rather dense 19-μm-thick TiTe_3_O_8_ films are prepared with the presence of a thin (<200 nm) reaction interlayer of Al_2_TeO_6_.

The dielectric properties of two most homogeneous and single phase TiTe_3_O_8_ thick films on Al_2_O_3_ substrates analysed by SPDR method at 20 GHz are presented in Table 1. 21-µm-thick TiTe_3_O_8_ films sintered at 680 °C for 5 h display a ε_r_ value of 28 and a Q × f value of 21,978, while 19-µm-thick films sintered at 700 °C for 5 h display ε_r_ of 32 and Q × f of 21,053. Thus, relative permittivity of TiTe_3_O_8_ thick films varies between 28 and 32 and Q × f varies approximately between 21,000 and 22,000. This uncertainty reflects the differences in the density, thickness and possibly the residual presence of the interfacial reaction with Al_2_O_3_. Indeed, the dielectric permittivity of the Al_2_TeO_6_ interface layer present in films sintered at 700 °C is smaller than that of TiTe_3_O_8_ [33]. In a series connection, this should deteriorate the total dielectric properties. However, the negative effect that may be induced by a <200 nm-thick interface layer is apparently compensated by higher density of the films sintered at 700 °C comparing to that of the films sintered at 680 °C.

The dielectric performance of TiTe_3_O_8_ films on Al_2_O_3_ substrates summarized in Table 1 is also compared to that reported for corresponding ceramics at high (GHz) frequencies. It is worthwhile to mention that Q × f for TiTe_3_O_8_ thick films have not been reported so far. ε_r_ of 50 and Q × f of 30,600 GHz at 5 GHz were reported for TiTe_3_O_8_ ceramics sintered at 720 °C [15]. Besides the lower measurement frequency, the observed differences of the dielectric behaviour between TiTe_3_O_8_ thick films and ceramics might also be related to density, residual presence of Al_2_TeO_6_ secondary phase and details of the microstructure. For other TiTe_3_O_8_ ceramics, sintered at 700 °C/5 h, ε_r_ and Q at 4 GHz were reported to be 36 and 3400, respectively [14]. Comparing these TiTe_3_O_8_ ceramics with our films on Al_2_O_3_, the results here obtained are rather promising. Moreover, the low losses of polycrystalline alumina (ε_r_ = 9, tanδ = 9.46 × 10^−5^, Q = 10570—for > 99% pure Al_2_O_3_) [34] should improve the dielectric performance of the final device TiTe_3_O_8_/Al_2_O_3_ composite structure.

Overall and by comparison, EPD TiTe_3_O_8_ thick films on Al_2_O_3_ reveal a high performance in terms of permittivity and loss tangent (high Q features) making them promising low-sintering-temperature dielectrics for micron sized high-frequency devices.

## 4. Conclusions

We have fabricated low-loss (high-Q) TiTe_3_O_8_ thick films directly on Al_2_O_3_ substrates by EPD and reported for the first time their microwave dielectric properties. Nineteen micrometre-thick TiTe_3_O_8_ films on Al_2_O_3_ sintered at 700 °C have the relative permittivity of 32 and Q × f > 21,000 GHz at a frequency of 20 GHz. Our results confirm the crucial role of a sacrificial carbon conductive coating on non-conductive Al_2_O_3_ substrate for the direct utilization of insulating substrates in EPD. During the sintering of TiTe_3_O_8_ films on Al_2_O_3_ at temperatures above 700 °C, an interfacial reaction between Al_2_O_3_ and TiTe_3_O_8_ takes place resulting in the formation of a stable aluminium tellurite compound, Al_2_TeO_6_. However, our findings also suggest that this thin (100–200 nm) interlayer of low-loss aluminium tellurite has no significant negative effect on the final properties of TiTe_3_O_8_ films. Finally, our results provide relevant information for the use of TiTe_3_O_8_ thick films on Al_2_O_3_ substrates as microlayer microstrip arrays in antennas, filters and transceivers for microwave telecommunications, satellite broadcasting and intelligent transport systems.

## Figures and Tables

**Table 1:** Relative dielectric permittivity, quality factor–frequency product and losses of TiTe_3_O_8_ thick films and ceramics. Sintering conditions are also indicated.

TiTe_3_O_8_		Dielectric Properties			Ref.
Type	Sintering Conditions	Measurement Frequency, GHz	Ε_r_	Q × f, GHz	
21-μm-thick TiTe_3_O_8_ films on Al_2_O_3_	680 °C/5 h	20	28	21,978	This work
19-μm thick TiTe_3_O_8_ films on Al_2_O_3_	700 °C/5 h	20	32	21,053	This work
TiTe_3_O_8_ ceramics	700 °C/5 h	4	36	13,600	[14]
TiTe_3_O_8_ ceramics	720 °C	5	50	30,600	[15]

## Data Availability

The data presented in this study are available on request from the corresponding author.
